# Two Cases of Malignant Tumor Affecting the Breast, and Their Treatment; with Some Remarks upon Malignant Growths in General*Read at the meeting of the Georgia State Medical Association, April, 1897.

**Published:** 1897-08

**Authors:** M. B. Hutchins

**Affiliations:** Atlanta, Ga., Clinical Lecturer on Dermatology and Syphilology, Atlanta Medical College


### TWO CASES OF MALIGNANT TUMOR AFFECTING THE BREAST, AND THEIR TREATMENT; WITH SOME REMARKS UPON MALIGNANT GROWTHS IN GENERAL.*

By M. B. HUTCHINS, M.D., Atlanta, Ga.,

Clinical Lecturer on Dermatology and Syphilology, Atlanta Medical College.

Although a great deal is being written about malignant disease of the mammary gland and its treatment, I do not remember any paper or discussion in the Georgia Medical Association upon this subject.

I report these cases not as anything new in pathology or brilliant in treatment, but with the hope of bringing out some general discussion among the members, many of whom are doing excellent work in this branch of surgery.

The operations and after-treatment were carried out, so far as practicable, as directed by Senn in his latest work on tumors. In preparing for any operation I have made it a rule to make works on anatomy my first reference, thoroughly familiarizing myself with the minutest details of the anatomy of the parts, thus avoiding the danger of having forgotten some essential point in the structures to be operated upon.

The anatomy is the first requisite; the technique can be obtained from the writings of those of more experience in a particular field, while the special details of one's own cases can be worked out on general principles.

Ideal cases, ideal patients, and ideal results do not always harmoniously come together. The duration and location of the disease, the circumstances and future of the patient, all combine to prevent our doing as we wish, and may even render impossible the carrying out of the best form of treatment. This accounts for many operations being done in a manner different from the classic method. In my first case I hesitated to perform an operation which

* Read at the meeting of the Georgia State Medical Association, April, 1897.

would prevent the patient doing the manual labor which was necessary to her existence, reserving the destruction of muscles to such time as it was no longer avoidable, should that time come.

*Case 1.*—Mrs. A. G., colored, aged thirty-eight, occupation housework and washing. Carcinoma, "scirrhus," of the right mammary gland. She first noticed a "small lump" in the right breast, just above and to the right of the nipple. She stated that her husband had struck her there at different times. She has had one child. No abscess of the breast. Examination showed a hard, rather well-defined tumor, the size of a small orange, in the middle and upper part of the gland. The upper part of the nipple was slightly retracted. Occasional stinging sensations were experienced. I could feel no enlargement of the glands, either at the lower border of the muscle or in the axilla. The patient weighed 175 pounds and was in robust health. She had quite a mustache and chin beard. Drs. L. P. Stephens and J. L. Campbell assisted at the operation. Every antiseptic precaution was employed. Elliptic incisions were made well away from the perceptible borders of the tumor, in the healthy skin, from the narrow part of the pectoralis major to the sternum and down to the pectoral sheath. The sheath of the muscle and mammary gland were removed by a clean dissection, no tearing, leaving a clear wound with only the muscle fibers as its floor. There was no perceptible gland at the lower edge of the muscle, nor could any be felt in the axilla. The incision was done in less than ten minutes.

There was remarkably little hemorrhage. Redundant fat under the edges of the incisions was trimmed away with scissors, then the flaps were made with the knife. An inch-incision was made in the lowest part of the gland cavity for drainage. It was necessary to ligate but one artery in the whole wound—hot water and torsion controlling the rest of the bleeding. The wound was brought together with four deep tension sutures of silkworm gut, about an inch apart. Then a continued suture of catgut was made from the outer end to near the inner, where a small opening was left for drainage with ten per cent. iodoform gauze. The line of suture was covered with iodoform one part, acid boric five parts, then many layers of ten per cent. iodoform gauze, salicylated cotton, and three wide bandages were applied; the arm being included finally, and additional support given the wrist with a towel.

Two days later the gauze drain below was removed and the incision closed with one suture. The outer dressings being stained, they were replaced with new ones. Five days after the operation all dressings were removed under strict antisepsis. The surface was perfectly clean, and the wound seemed to have healed *per primam*, except at the outer end and at the internal drainage opening. The gauze was removed from the latter, being followed by about half an ounce of bloody fluid. After applying pressure to remove any remaining fluid, another piece of gauze was inserted. The lower drainage incision was granulating nicely, with a little oozing of bloody serum visible. Dressings were made as at the time of operation.

Eight days after the operation the inner drainage and the four tension sutures were removed. There was perfect primary union save at the two extremities. The suture was removed from the lower drain incision also. The wound was clean and healing. The surface was covered with a lighter dressing and the arm left free. Two days later only the lower drain remained unhealed. Gauze was applied there and cotton to the rest, with oblique adhesive strips.

Healing was complete in the lower drain eighteen days after the operation. There was never any pus. At the inner end of the main incision there was a lump of compressed fat.

The patient was seen nearly four months after the operation, for the last time. The inner half of the united wound showed perfect union, the outer half a faint line of cicatrix. A small cicatrix marked the site of the lower drain. There was absolutely no sign of recurrence, nor of glandular involvement. The patient left the city and I have not seen her again. I was informed that she remained in good health.

The operation was done in this case with full knowledge of its probable incompleteness, this being due, in the first place, to the wishes of the patient and to the fact that it was preferable to complete it at a later time if it should become necessary. Four months later that time seemed not yet to have arrived. Under similar circumstances, I should be again willing to risk dividing the operation.

In spite of the fact that the microscope shows very early gland involvement in the majority of cases, it is possible, in a scirrhous growth like this, for metastasis not to have occurred at the time of operation.

*Case 2.*—Mrs. P., aged forty-six, a farmer's wife. Sarcoma of the right upper pectoral region and the upper part of the mammary gland. About December, 1893, she felt a burning sensation in the skin of the chest over about the fourth rib. The spot also looked as if burned. It next took on the appearance of a boil, or "rising," of normal skin color, save in the center, where was a band, about one-half by one inch in dimensions, of purplish color. The growth was hard to the touch, its borders well defined.

It gradually enlarged until April 28, 1896, to a roundish, small hen egg-sized tumor of the skin and subcutaneous tissues. The overlying skin had been involved from the beginning. The only discolored part was still the perpendicular, purplish band across its center. The middle of a line between the anterior border of the right axilla and the center of the sternum would mark the central part of the growth. Sensations of a burning character had been present from the beginning—in fact, this feeling first attracted her attention to the disease. The burning was worse when she was exposed to heat. She first thought it was a burn from hot grease, but this was not possible, through the fact that her clothing covered the part. She remembered no injury to this region.

On April 28, 1896, her physician excised everything which he thought diseased. The mass removed weighed eight to ten ounces and was the size of a small fist. Two stitches were used to close the elliptical wound, but they did not hold. At the first dressing there was pus, and then an attempt was made to obtain healing by granulation. But the disease soon reasserted itself, showing a red areola and becoming decidedly active about four months later. The fungoid appearance dates from this time, her friends said, corresponding with a change in the form of dressing.

At the time of my examination, about two months later, there was in the center of the right pectoral region a firm, rounded, smooth mass with hard base; the edges near an inch thick, the skin adherent. It was four by four inches in actual size, apparently movable over the pectoralis major, but continuous with the upper segment of the mammary gland. The skin over its edges was from a deep red to a purple color. In the center, comprising about one-third of the growth, it was elevated about one inch over the rest of its surface. This elevated part was irregular, papillomatous, cauliflower-like, fungoid and grayish, with thin, whitish, purulent fluid between the masses. In the center of this there was an irregular opening, apparently as deep as the growth, and of the size of a walnut. At the upper edge of the fungous part was a finger-tip-sized, carbuncle-like lesion. These were said to be frequent and of short duration. The fungoid mass had overhanging edges. Now and then she experienced a "shooting pain" towards the axilla. In spite of antiseptic dressings, there was a decided odor. The lymphatic vessels and glands towards the axilla could not be felt.

When the first disease appeared she weighed nearly two hundred pounds, but since then her weight had gone down to one hundred and fifty or one hundred and sixty pounds. The menopause occurred after the excision of the first tumor. She contracted a cold two months before my examination and had a cough, dating from that time, without chest pain, the sputum being of light color and thick. Lung resonance seemed perfect and the pulse normal. There was no appearance of cachexia, and she had done her housework for most of the time. I could discover no evidence of internal metastasis.

The night before the operation I shaved the axilla and scrubbed the field of operation with soap, ether and bichloride solution. The fungoid part was cleansed with peroxide of hydrogen; ten per cent. iodoform gauze was packed in the opening, and spread over the whole area, being held by a bandage.

Drs. Jones and Bates, of Murray county, assisted at the operation. The room having been heated, the patient was stripped to the waist, and the anesthesia begun, after the ordinary hypodermic dose of morphia and atropia. Four ounces of chloroform were used and the operation then completed under ether, the chloroform being used at first on account of the cough. The entire field of operation was cleansed as the evening before. The instruments and everything which would touch them or the wound were thoroughly sterilized. A preliminary incision was made curving downward about an inch below the nipple, from the center of the sternum to the anterior border of the axilla. Then a second incision—curved at least an inch above the border of the tumor, and extending near to the clavicle—was made from the center of the sternum to meet the first at the axillary border. The mammary gland and the tumor mass were then separated from the pectoralis major and left attached only near the axilla. The incision was then carried along the lower edge of the tendon of the muscle to near its insertion at the humerus. After reflecting the skin and fat, a director and the fingers were worked down to the axillary artery and vein. The entire axilla was then thoroughly cleared out, with, I regret to say, a little more tearing than cutting. A few bean-sized glands of livid color were found here, but none at the lower border of the muscle.

All spurting arteries were caught with forceps, being tied later, if necessary, with sterilized catgut. As the center of the diseased mass was found to have involved the pectoralis major, this muscle was now entirely removed from the chest wall, leaving only a narrow part along the edge of the deltoid and the tendon. As the pectoralis minor appeared healthy it was not disturbed. (The patient having been chilled, was given a little whisky hypodermically.) The operation was completed in an hour and forty minutes, suturing and dressing requiring about three quarters of an hour more. Before closing the wound an opening about two inches long was made at the bottom of the pocket left by the removal of the gland, at the insertion of the serratus magnus. A sheet of ten per cent. iodoform gauze was laid over the wound, and one end folded and brought out at the drainage opening below.

The wound and adjacent skin were thoroughly cleansed, bleeding controlled by hot water and perhaps two vessels ligated. The skin and fat over the sternum were cut under for an inch or two, the edges of the wound were pushed together, and held with seven tension sutures of silkworm gut. Badly fitting margins of skin were cut away, and the entire wound margins were then brought in apposition with a continued suture of catgut.

The line of union as well as the incision for drainage was covered with iodoform one part, acid boric five parts; then many layers of iodoform gauze were applied, and over all an abundance of salicylated cotton, and finally about twenty-four yards of three-inch bandage to hold the dressings and confine the arm. The patient reacted well.

The operation was completed about noon and I left early the next morning. I heard nothing from the patient for exactly three months. Then the doctor wrote that she was apparently sound and well. He had removed the gauze drain the second day and the dressings on the seventh day. He says he found a half pint of pus under the dressings, but her temperature had never been over 100 degrees nor her pulse over 90—a remarkable circumstance. At any rate, he wrote that a part of the margins were lost and the corresponding portion of the wound healed by granulation. The patient complained only of a little tension on that side.

This case was operated upon in a farmhouse in Murray county. The air and surroundings were good. I carried to the place, everything which could be needed, save the water and fire, and the doctors who assisted me brought nothing. I confess that I did not clear out the axilla in the best manner, by pure dissection, but as sarcoma does not so freely affect the glands, and was as likely to have gone elsewhere as in the direction of the axilla, I do not think I did any harm. Had I left any sarcoma cells the result would have been like that after the first operation.

There is no longer any doubt among well-informed physicians that malignant growths are at first purely local, and not constitutional in the sense that they can be cured by internal therapeutic measures. The carcinomata take their origin from cells which belong to the epiblastic or to the hypoblastic structures, in other words they are of epithelial origin. The sarcomata are growths of mesoblastic origin; they belong to the connective tissue structures.

Benign tumors show no tendency to the separation, migration and transplanted growth of their elements. Such a course is characteristic of malignant growths, in fact this feature of metastasis constitutes their malignancy. Many benign tumors are encapsulated or well defined, and a cure is had by ordinary excision. Malignant tumors cannot be so easily eradicated, because of their tendency to metastasis, unless the excision is done before metastasis beyond the visible borders has occurred. Malignant growths have no wall nor capsule, even the apparent capsule of a sarcoma showing evidence of the disease, under the microscope.

Absolute removal or destruction of a malignant growth insures its cure, but this treatment may either be impossible, or the surgeon may make the mistake of operating as for a benign growth. Not only must the tumor with a large margin of apparently healthy tissue be removed, but every particle of possibly diseased tissue must also be included in the mass. For example, if the lower lip be involved and the glands beneath the jaw, the lip, as widely as necessary, and all the tissue between it and a point below the diseased glands must be excised in one piece. If it be a simple-appearing sarcoma of the foot or hand and we are not absolutely sure that it is yet local, amputation had best be done at once, rather than wait until amputation must follow amputation and death follow at last.

There is no excuse for a visibly epitheliomatous lesion of the penis being treated by simple amputation, if the inguinal glands are involved, and then skipping over and removing the diseased glands. If cells have traveled along the lymph channels to gain access to the glands, it is more than likely that others are in transit when we operate. If we leave the infected channels, we do not treat the case properly or successfully. What is necessary in mammary cancer is necessary in cancer of any other part.

A point which McClellan brings out in his "Regional Anatomy" is apparently overlooked by many surgeons. The lymphatics which lead from the inner segment of the mammary gland pass between the ribs into glands beneath the sternum. Now, if the disease involves the inner part of the breast it seems reasonable that metastasis should take place towards the substernal region, and thence upward, along the internal mammary vessels, to the subclavian region. Then, there is a superficial lymphatic channel from the nipple to a gland beneath the outer border of the clavicle. Such being the case, an operation devoted entirely to the region between the sternum and the apex of the axilla, thence upward under the clavicle, may leave untouched an active focus of disease. Until every point of lodgment for cancer cells is removed the treatment falls short of success.

An English surgeon has lately stated that the mammary carcinomata shows early metastasis to the bone marrow. If he is correct there is but one chance of successful treatment, and that is an important element to have in any case, namely, early diagnosis and treatment.

As regards skin metastasis and so-called recurrences, there are cases in which neither of these factors seems sufficient to explain the condition present. This applies specially to skin cancer. We know that multiple epitheliomata do occur on the skin. It is also a fact that both mammary glands may be affected with carcinoma. The possibility that more than one focus of disease can exist should not be forgotten, and all cases which show the disease at other points than those treated should not be set down as having originated from the one diseased center. If a person has a carcinomatous tendency he can develop more than one, each independent of the other.

This statement is made with the knowledge that the majority of carcinomata do originate from one original focus, but there are certain cases in which the surgeon should not be held responsible for another cancerous development any more than he should be charged with the occurrence of the disease in some one else.

311-312 *Fitten Building.*

